# Sickle Cell Disease Activates Peripheral Blood Mononuclear Cells to Induce Cathepsins K and V Activity in Endothelial Cells

**DOI:** 10.1155/2012/201781

**Published:** 2012-04-09

**Authors:** Philip M. Keegan, Sindhuja Surapaneni, Manu O. Platt

**Affiliations:** Wallace H. Coulter Department of Biomedical Engineering, Georgia Institute of Technology and Emory University, Atlanta, GA 30332, USA

## Abstract

Sickle cell disease is a genetic disease that increases systemic inflammation as well as the risk of pediatric strokes, but links between sickle-induced inflammation and arterial remodeling are not clear. Cathepsins are powerful elastases and collagenases secreted by endothelial cells and monocyte-derived macrophages in atherosclerosis, but their involvement in sickle cell disease has not been studied. Here, we investigated how tumor necrosis alpha (TNF*α*) and circulating mononuclear cell adhesion to human aortic endothelial cells (ECs) increase active cathepsins K and V as a model of inflammation occurring in the arterial wall. ECs were stimulated with TNF*α* and cultured with peripheral blood mononuclear cells (PBMCs) from persons homozygous for sickle (SS) or normal (AA) hemoglobin. TNF*α* was necessary to induce cathepsin K activity, but either PBMC binding or TNF*α* increased cathepsin V activity. SS PBMCs were unique; they induced cathepsin K in ECs without exogenous TNF*α* (*n* = 4, *P* < 0.05). Inhibition of c-Jun N-terminal kinase (JNK) significantly reduced cathepsins K and V activation by 60% and 51%, respectively. Together, the inflammation and activated circulating mononuclear cells upregulate cathepsin activity through JNK signaling, identifying new pharmaceutical targets to block the accelerated pathology observed in arteries of children with sickle cell disease.

## 1. Introduction

 Sickle cell disease is a genetic disorder that causes *in vivo* polymerization of hemoglobin molecules into rigid fibers within red blood cells, deforming them in the canonically described “sickle” shape. Rigid, sickled red blood cells and the byproducts of their hemolysis cause chronic vascular damage and increase systemic levels of inflammatory cytokines, mobilized mononuclear cells [1], and pathological levels of increased monocyte adhesion to the endothelium [2, 3]. Overall, these pathological inflammatory conditions and mononuclear cell-endothelial cell interactions may contribute to intimal thickening, and lumen narrowing seen in pulmonary hypertension and stroke lesions of children; pulmonary hypertension is responsible for 20–30% of sickle-cell-related deaths in adult patients [4, 5] and 11% of children with sickle cell disease will suffer from a major stroke by the age of 16.

Both of these clinical syndromes are characterized by vascular remodeling [6–8]. Vascular remodeling analogous to stroke lesions in sickle cell disease has been observed in atherosclerosis, the major cardiovascular disease, where mononuclear cell infiltration of the subendothelial space, degradation of the elastic lamina, and subsequent smooth muscle cell proliferation mediate lesion progression and luminal narrowing [2]. These similarities suggest that common mechanisms for arterial remodeling may exist between the well-studied, well-characterized atherosclerosis, and the less understood mechanisms of sickle cell disease.

 Arterial remodeling can be defined as changes in the composition of proteins, cell types, and even cell phenotypes that induce chronic effects on the structure, mechanical properties, and total health of the artery [6–8]. This includes degradation of old matrix by newly activated proteases as well as synthesis and deposition of new extracellular matrix proteins. Cysteine cathepsins, one such family of proteases upregulated in arterial remodeling [6, 9], belong to the papain superfamily of proteases and contain the most potent human collagenases and elastases [10]. Increased cathepsin activity has been linked to tissue destruction in the cardiovascular system with atherosclerotic elastic lamina degradation [11–13], stent restenosis [14, 15], abdominal aortic aneurysm formation [16], and heart valve remodeling under hypertensive conditions [9].

 Two cathepsins in particular have gained significant interest in their role in arterial remodeling in cardiovascular disease. Cathepsin K is the most potent human collagenase yet identified [17], as well as an extremely powerful elastase [18, 19]. Additionally, cathepsin K has been shown to be highly expressed in atherosclerotic lesions where it degrades arterial collagen and subendothelial elastic lamina [12, 13]. Cathepsin V is the most powerful mammalian elastase yet identified and is expressed in human monocyte-derived macrophages [10]. Studies have shown that the human cathepsin V homolog, murine cathepsin L [20, 21], significantly contributes to cardiovascular disease in mouse models [9, 22]. Neither of these two enzymes has been linked to sickle-cell-disease induced vascular wall remodeling and pathology.

 In this study, we evaluated the potential involvement of cathepsin-mediated arterial remodeling in sickle cell disease by studying the effects of TNF*α* stimulation and adhesion of mononuclear cells isolated from whole blood of individuals homozygous for the sickle mutation on endothelial cell expression and activation of cathepsins K and V. We employed a novel, multiplex cathepsin zymography technique to simultaneously quantify the active forms of cathepsins K, L, S, and V in response to the different stimulation and coculture conditions [23]. Furthermore, we investigated the phosphorylation of key kinases to identify intracellular signaling cascades linking TNF*α* stimulation and mononuclear cell binding to increased levels of active cathepsins K and V as a proposed model for the unique and accelerated tissue remodeling observed in arteries of children and adults living with sickle cell disease.

## 2. Materials and Methods

### 2.1. Ethics Statement

All protocols were reviewed and approved by the Georgia Institute of Technology Institutional Review Board, and informed consent was received from all participants. In the case of minors, assent was provided by parents/guardians.

### 2.2. Cell Culture

Human aortic endothelial cells (HAECs) (Lonza) were cultured in MCDB medium 131 (Mediatech) containing 10% fetal bovine serum (FBS), 1% L-glutamine, 1% penicillin/streptomycin, and 1% endothelial cell growth serum (ECGS). Cells were maintained with 5% CO_2_ at 37°C.

### 2.3. TNF*α* ELISA

Whole blood samples were allowed to coagulate for 6 hours, followed by centrifugation at 900 g for 30 minutes to remove platelets and cells. The supernatant was collected, and TNF*α* levels were quantified using an enzyme-linked immunosorbent assay (ELISA) specific for soluble, human TNF*α* (R&D Biosystems). Absorbance values were recorded using Synergy 4 (Biotek) at 450 nm with correction readings at 540 nm. Quantification of TNF*α* protein levels was calculated by generating a four-parameter logistic standard curve using Gen5 software (Biotek).

### 2.4. Peripheral Blood Mononuclear Cell Isolation

Whole blood samples were obtained from males and females homozygous for sickle (SS) or normal (AA) hemoglobin; patients on hydroxyurea, chronic transfusion, or who had experienced a recent crisis were excluded from this study. Whole blood samples were centrifuged against a Ficoll-Paque density gradient (density: 1.077 g/mL; GE Healthcare) for 30 minutes at 2450 rpm to separate the buffy coat layer. After centrifugation, peripheral blood mononuclear cells (PBMCs) were aspirated, washed in PBS, and pelleted by centrifugation for 10 minutes. The isolated cells were then washed with a red blood cell lysis buffer (0.83% ammonium chloride, 0.1% potassium bicarbonate, and 0.0037% EDTA) for seven minutes to remove any contaminating RBCs. Cell number and viability were determined using a Vi-Cell (Beckman Coulter).

### 2.5. PBMC Adhesion Assay

HAECs were preconditioned in normal growth media in the presence or absence of 10 ng/mL recombinant human TNF*α* (Invitrogen) and cultured for 4 hours prior to the addition of 500,000 PBMCs/mL. Isolated PBMCs were allowed to adhere for 45 minutes prior to washing three times with PBS, and then cocultures were maintained for an additional 20 hours. For JNK inhibition studies, endothelial cells were preconditioned with 10 *μ*g/mL of SP600125 (EMD Biosciences) for one hour prior to addition of media containing vehicle, 10 ng/mL TNF*α*, and/or 10 *μ*g/mL of SP600125.

### 2.6. Phosphorylated Kinase Screening

Cell lysates were prepared per BioPlex Suspension Array System instructions (BioRad). Lysates were incubated overnight with fluorescently labeled beads specific for the phosphorylated forms of Akt (Ser473), extracellular signal-regulated kinases 1 and 2 (Thr202/Tyr204, Thr185/Tyr187), c-Jun NH_2_-terminal kinase (JNK) (Thr 183 /Tyr 185), and c-Jun (Ser63) (BioRad). The samples were then washed and incubated with kinase-specific, biotinylated antibodies for 2 hours, followed by treatment with avidin/streptavidin tagged with phycoerythrin. Phosphorylated kinase levels were measured using a BioPlex 200 System (BioRad).

### 2.7. Multiplex Cathepsin Zymography

Cathepsin zymography was performed as described previously [24]. Determination of cathepsin V band required incubation in acetate buffer, pH 4 [25]. Gels were imaged using an ImageQuant 4010 system (GE Healthcare). Images were inverted in Adobe Photoshop and densitometry was performed using Scion Image.

### 2.8. Statistical Analysis

Each experimental condition was repeated with a minimum of three biological replicates, and each data point is presented as the mean value and standard error of the mean. Representative images are shown. Unpaired student *t*-tests were used to determine statistical significance (*P < 0.05) between most experimental groups.

## 3. Results

### 3.1. Sickle Cell Disease Preconditions Circulating PBMCs to Induce Cathepsin K Activity

**Figure 1:**
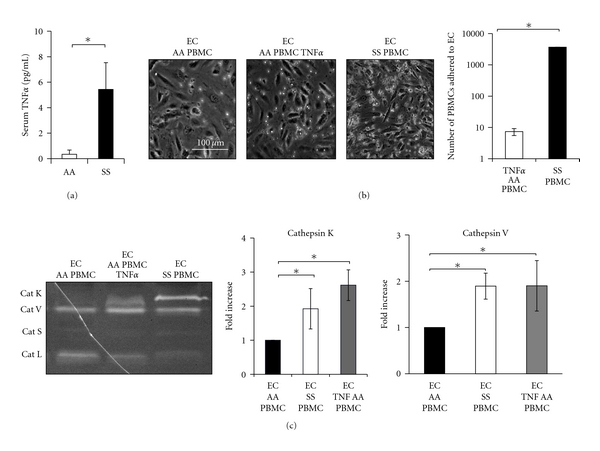
Sickle cell disease preconditions circulating peripheral blood mononuclear cells to induce cathepsin K activity. Whole blood samples were obtained from donors homozygous for the normal *β*-globin allele (AA) and homozygous for the sickle allele (SS). (a) Baseline serum levels of TNF*α* were quantified using an ELISA specific for human TNF*α* (*n* = 3, *P < 0.05, SEM bars shown). (b) PBMCs were isolated via differential centrifugation through a density gradient. For cocultures, confluent EC cultures were preconditioned with 10 ng/mL TNF*α* for 4 hours, prior to the addition of either AA or SS PBMCs. Nonadherent cells were washed away, and cocultures were maintained for an additional 20 hours. Representative images of cocultures were used for mononuclear cell adhesion counts. (c) Cells were lysed and cathepsin K activity was assessed using multiplex cathepsin zymography and quantified via densitometry (*n* = 10, *P < 0.05).

 Whole blood samples were obtained from donors homozygous for normal (AA) or sickle (SS) hemoglobin. First, an ELISA was run to quantify blood serum levels of TNF*α*. SS donors had 5.43 ± 2.3 pg/mL of TNF*α* compared to 0.3 ± 0.3 pg/mL of TNF*α* in AA controls (*n* = 3, *P* < 0.05), an almost 20-fold increase (Figure 1(a)). TNF*α* stimulation of endothelial cells increased the adhesion of AA PBMCs, compared to unstimulated EC cultures (Figure 1(b)); however, the number of adhered SS PBMCs was 100 times higher than TNF*α* stimulated AA PBMC cocultures (Figure 1(b); *n* = 3, *P* < 0.001). Cells were cultured together for an additional 20 hours for cathepsin induction, prior to lysing, collection, and multiplex cathepsin zymography. SS PBMCs significantly increased levels of active cathepsins K and V when cocultured with endothelial cells, and without exogenous TNF*α* stimulation (Figure 1(c)), suggesting that the SS PBMCs were preconditioned to induce this activity. AA PBMC cocultures in the absence of TNF*α* lacked detectable bands of active cathepsin K (Figure 1(c), left lane).

### 3.2. TNFa Stimulation and PBMC Interactions with Endothelial Cells Activate JNK Signaling

**Figure 2:**
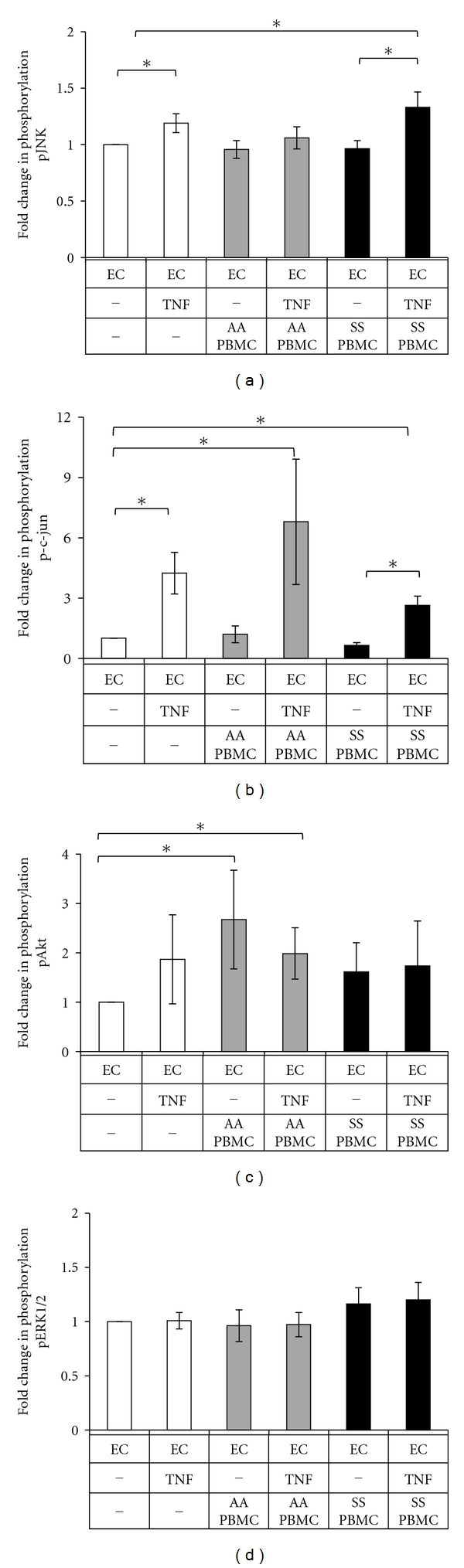
TNF*α* and PBMC interactions increase JNK and Akt phosphorylation. Confluent HAECs were cocultured with peripheral blood mononuclear cells isolated from AA or SS donors, and lysates were collected for kinase analysis. Levels of phosphorylated (a) JNK, (b) c-Jun, (c) Akt, and (d) ERK1/2 were measured, and phosphorylated kinase signals were normalized to unstimulated HAEC control (*n* = 3, *P < 0.05, SEM bars shown).

 To investigate the intracellular signal cascades increasing the levels of active cathepsins K and V downstream of TNF*α* and PBMC adhesion cues, we measured phosphorylation of JNK, c-jun, Akt, and ERK1/2 using Bioplex/Luminex technology, a quantitative bead-based immunofluorescent assay that allowed measurement of all four signals in one cell extract after 24 hours of coculture. JNK and its downstream signaling protein substrate, c-Jun, showed the greatest activation in response to TNF*α* stimulation with or without AA or SS PBMCs (Figures 2(a) and 2(b), *n* = 3, *P* < 0.01) with c-Jun activation as high as 6-fold that of the EC controls. Akt phosphorylation was significantly increased by AA PBMC binding alone even without TNF*α* stimulation (Figure 2(c), *n* = 3, *P* < 0.01). There were no changes in ERK 1/2 phosphorylation in any condition for all time points measured (Figure 2(d)).

### 3.3. Cathepsins K and V Activities Induced by Sickle Cell Disease PBMCs Were Significantly Reduced by JNK Inhibition

**Figure 3:**
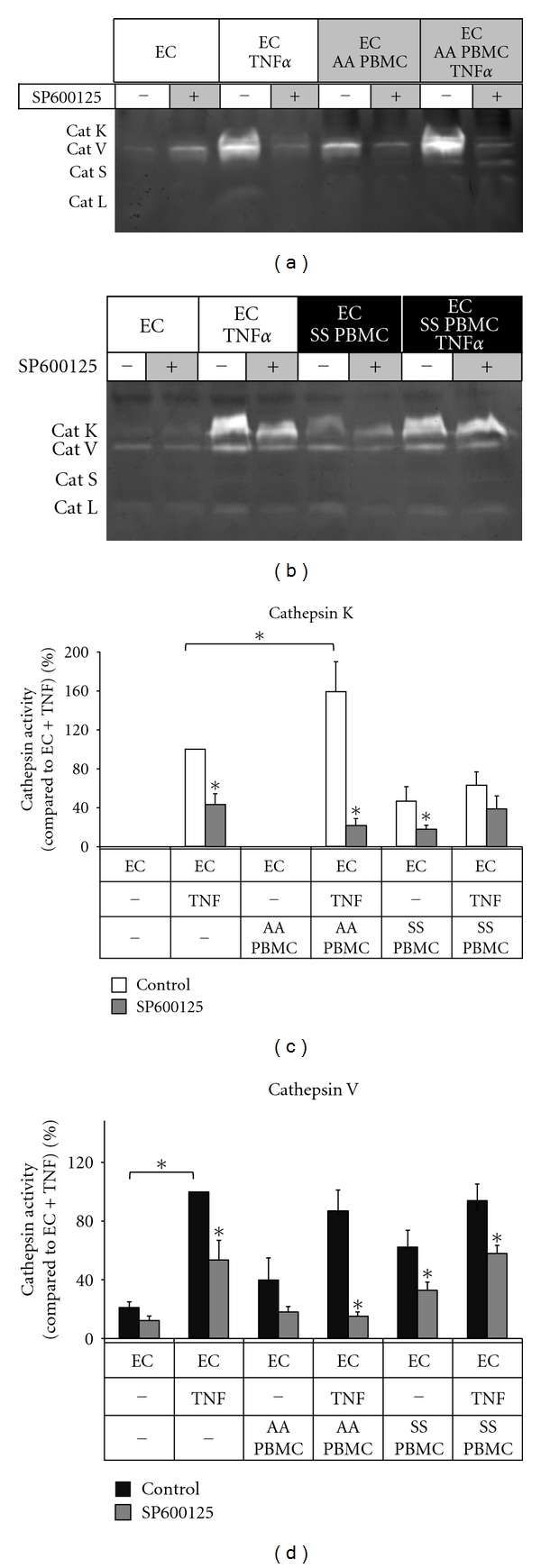
Cathepsins K and V activities induced by sickle cell disease PBMCs are significantly reduced by JNK inhibition with SP600125. HAECs were incubated with or without 10 *μ*M of the JNK inhibitor, SP600125, 1 hour prior to TNF*α* stimulation, as described previously. Cocultures with AA or SS PBMCs were maintained for an additional 20 hours. Cell lysates were collected and analyzed via multiplex cathepsin zymography. Densitometric analysis quantified active cathepsins K and cathepsin V (*n* = 3, *P < 0.05, SEM bars shown).

 Since JNK and c-jun phosphorylation were significantly upregulated, we tested if inhibiting this signal cascade would block the increase in levels of active cathepsins K and V by endothelial cells after adhesion and coculture with SS PBMCs. HAECs were cultured with or without SP600125, a JNK inhibitor, for 1 hour prior to addition of 10 ng/mL TNF*α* or vehicle. AA or SS PBMCs were subsequently added, and nonadhered cells were washed away. Cell lysates were collected after 24 hours, and cathepsin activity was assessed through multiplex cathepsin zymography. SP600125 significantly reduced the upregulated cathepsin K and cathepsin V activities of unstimulated SS PBMCs when cocultured with endothelial cells by 48% and 29%, respectively (Figure 3; *n* = 5, *P* < 0.05). 

## 4. Discussion

 Endothelial cell expression of cathepsins and increased cathepsin-mediated elastase activity are upregulated during atherosclerotic development and induced by inflammation and altered hemodynamics [9, 12, 13, 26, 27], which are both present in sickle cell disease [26], leading to our hypothesis that elevated TNF*α* and increased circulating mononuclear cells would stimulate increased endothelial cell cathepsin activity. This elevated activity may contribute to arterial remodeling in sickle cell disease. The findings of this study specifically implicate TNF*α* and mononuclear cell binding to endothelium as key mediators, and that circulating mononuclear cells in sickle cell disease are predisposed to induce cathepsin proteolytic activity.

 Here, we have specifically shown that TNF*α* stimulation increased the expression and activity of the most potent mammalian collagenase and elastase, cathepsins K and V, respectively (Figure 1). Additionally, SS PBMCs significantly increased cathepsin K activity in endothelial cells in the absence of TNF*α*, suggesting that they were preconditioned in the blood for adhesion to endothelium and cathepsin K induction (Figure 1); AA PBMCs required TNF*α* stimulation to reach these higher levels of cathepsin K and V (Figure 1). These findings are consistent with reports that circulating sickle erythrocytes increase mononuclear cell activation and adhesion to endothelial cells [28] and support our hypothesis that the blood milieu of people living with sickle cell disease predisposes circulating mononuclear cells to adhere to endothelium and promote arterial remodeling. Previous studies have already established that the circulatory environment in sickle cell disease preconditions peripheral blood mononuclear cells into a pathologically activated state, where these cells produce 139% more TNF*α* per cell than control mononuclear cells [28, 29]; these mechanisms may be at play here leading to increased active cathepsins K and V.

 Inhibition of JNK signaling with SP600125 reduced the inflammation-induced activation of cathepsins K and V in AA and SS PBMC cocultures with endothelium (Figure 3). These findings highlight the role of JNK signaling as an integration control point and as a therapeutic target to inhibit the initiation of gene and protein expression in response to inflammatory stimuli resulting in endothelial cell upregulation of cathepsins K and V protein and activity. More importantly, the predisposition of SS PBMCs to induce these effects suggests that these novel mechanisms may be occurring constantly in the vasculature of individuals with sickle cell disease. It will be important to continue these studies quantifying cathepsin activation of SS donors with and without stroke or with high transcranial Doppler velocities known to be a risk factor for stroke to parse differential activation mechanisms potentially responsible for the increased risk. Such investigations may reveal novel biomarkers relevant to stroke risk prediction in pediatric patients and open new avenues for pharmaceutical therapies to prevent the arterial remodeling and luminal narrowing that cause cardiovascular complications and death.

## 5. Conclusion

 Elevated inflammatory factors and circulating mononuclear cells inherent to sickle cell disease induce pathologically high levels of cathepsins K and V activity when binding to and stimulating endothelial cells, increasing proteolytic activity that may be involved in arterial wall remodeling to increase risk of stroke and pulmonary hypertension. There is a pressing need for novel pharmaceutical targets to inhibit these activities, and from this work, we propose that JNK, cathepsin K, and cathepsin V are three new targets for inhibition to reduce pathological arterial remodeling in sickle cell disease.
